# Risk factors for extubation failure in the intensive care unit

**DOI:** 10.5935/0103-507X.20180046

**Published:** 2018

**Authors:** Aracely Lizet Silva-Cruz, Karina Velarde-Jacay, Nilton Yhuri Carreazo, Raffo Escalante-Kanashiro

**Affiliations:** 1 Facultad de Ciencias de la Salud, Universidad Peruana de Ciencias Aplicadas - Lima, Perú.; 2 Unidad de Cuidados Intensivos, Instituto Nacional de Salud del Niño - Lima, Perú.

**Keywords:** Risk factors, Airway extubation, Respiration, artificial, Intensive care units, pediatric

## Abstract

**Objective:**

To determine the risk factors for extubation failure in the intensive care unit.

**Methods:**

The present case-control study was conducted in an intensive care unit. Failed extubations were used as cases, while successful extubations were used as controls. Extubation failure was defined as reintubation being required within the first 48 hours of extubation.

**Results:**

Out of a total of 956 patients who were admitted to the intensive care unit, 826 were subjected to mechanical ventilation (86%). There were 30 failed extubations and 120 successful extubations. The proportion of failed extubations was 5.32%. The risk factors found for failed extubations were a prolonged length of mechanical ventilation of greater than 7 days (OR = 3.84, 95%CI = 1.01 - 14.56, p = 0.04), time in the intensive care unit (OR = 1.04, 95%CI = 1.00 - 1.09, p = 0.03) and the use of sedatives for longer than 5 days (OR = 4.81, 95%CI = 1.28 - 18.02; p = 0.02).

**Conclusion:**

Pediatric patients on mechanical ventilation were at greater risk of failed extubation if they spent more time in the intensive care unit and if they were subjected to prolonged mechanical ventilation (longer than 7 days) or greater amounts of sedative use.

## INTRODUCTION

Mechanical ventilation (MV) is a necessary life support therapy for 30-64% of pediatric patients admitted to an intensive care unit (ICU).^(1)^ To provide invasive MV, it is necessary to have patients sedated with their airways secured with tracheal tubes.^(2,3)^ Prolonged MV can result in complications and side effects.^(4)^ The current literature recommends extubating patients as early as possible according to their clinical courses and when the causes that led to the need for ventilatory and/or oxygen support are reversed.^(2,5)^ However, extubation is not always successful, and the literature indicates that approximately 4.9 to 22% fail this process and require re-intubation.^(3,6-9)^

The process of extubation consists of the removal of the tracheal tube when the patient's physiological state recovers, which allows him to maintain spontaneous ventilation.^(3)^ To achieve this, there are protocols for ventilatory weaning. Weaning is the gradual reduction of ventilatory support, and it represents between 40 and 50% of the total time on MV. In this time, the patient is given spontaneous breathing time to attempt an acceptable gas exchange.^(10,11)^ The clinical criteria for weaning are based on the control or resolution of the cause of respiratory failure, an adequate gas exchange with a positive end-expiratory pressure (PEEP) ≤ 8cmH_2_O and a fraction of inspired oxygen (FiO_2_) ≤ 0.5, maintenance of spontaneous respiratory effort, suspension of sedation and muscle relaxants, the absence of clinical signs of sepsis, the presence of a cough reflex, and the correction of any metabolic and electrolyte imbalances. All of this is designed to guarantee the protection of the airway and a stable hemodynamic state.^(12-14)^

Failed extubation (FE) is considered to have occurred when reintubation or respiratory assistance is needed within 48 hours of a scheduled extubation.^(15)^ However, there are large differences in results between different investigations that have examined failed extubation rates.^(15,16)^ Unlike patients who achieve successful extubation, those with FE have high rates of morbidity and mortality. FE also prolongs the duration of MV and thus causes a longer stay in the ICU. This has as a consequence; with the longer resulting hospital stay, other complications increase, such as the need for tracheotomy, the incidence of pneumonia and pulmonary damage induced by MV (VILI), and finally, costs increase as well.^(8)^

At present, several pediatric studies have shown dissimilar results regarding the risk factors associated with FE.^(8,9,16-18)^ In a retrospective study in the United States, the associated risk factors were a young age, the use of MV for longer than 7 days, an oxygenation index of greater than 5, the use of vasopressors and the administration of intravenous sedative drugs for greater than 5 days.^(8)^

In a prospective and multicenter study, the risk factors for FE were found to be an age under 24 months, genetic syndromes, and respiratory and chronic neurological alterations.^(17)^ Additionally, a study conducted in Chile discovered an association between FE and the time on MV and the length of stay in the ICU.^(16)^ MV is classified as short-lived when the duration is less than three days and as long-term or prolonged when the duration is greater than seven days.^(16)^

It is considered important to identify and monitor the risk factors associated with FE in order to optimize the doctor's decision to extubate a pediatric patient undergoing MV. For this reason, the present study aims to determine the risk factors associated with FE in the pediatric ICU.

## METHODS

This is a case-control study carried out in the ICU of the *Instituto Nacional de Salud del Niño* (INS). It is the main referral hospital for pediatrics in this country, with excellent treatment and technological capabilities. For this research, a secondary database of patients hospitalized in the ICU between 2011 and 2015 was used as a reference. We proceeded with the collection of data from the medical records of the patients who were on MV. Then, we calculated the prevalence of patients on MV, as well as those with FE, using the hospital's secondary database.

The following inclusion criteria were considered: the first extubation of patients between the ages of 1 month and 17 years 11 months who were on MV for a time equal to or greater than 24 hours. The following exclusion criteria were also considered: patients with congenital malformations of the airway and events of multiple extubations. The same inclusion and exclusion criteria were used for both the cases and controls. In this study, cases were defined as those with FE, and controls as those with successful extubations (SE).

The present study was evaluated and approved by the Ethics Committee of the *Instituto Nacional de Salud del Niño* (INS) and the *Universidad Peruana de Ciencias Aplicadas* (UPC). Informed consent was not carried out because there was no contact with the patients, only with the data included in the database and the medical records. Although the population under study is vulnerable, it did not represent any risk for the patients because no type of intervention was carried out. The confidentiality and privacy of the patients was maintained through anonymization in order to respect their autonomy. The data were only handled by the researchers from this study and were strictly safeguarded, guaranteeing their safety.

The sample was calculated using the statistical package Epidat 4.1. The variable used was an MV duration of greater than 7 days, which was extracted from Cruces et al.^(16)^ The formula for case-studies and controls was used, with an expected odds ratio (OR) of 6.95, a confidence interval of 95% (95%CI), a power of 80% and a proportion of exposed cases of 20%. The calculation was made taking into account one case for every four controls. The resulting sample included 30 patients in group A (FE) and 120 patients in group B (SE). Non-probabilistic sampling was carried out for both the cases and controls, as out of the 44 cases, 30 met the criteria and were included. Likewise, the first 120 controls that met the inclusion criteria were included.

The main variables were FE and SE. FE was defined as reintubation or respiratory assistance being necessary within the first 48 hours after extubation. SE was defined as when the patient showed the ability to maintain spontaneous ventilation without respiratory support 48 hours after extubation.

The categorical variables were: length of MV of greater than 7 days, sex, reason for hospital admission (respiratory or not), use of sedatives by infusion for greater than 5 days, use of inotropes for greater than 10 days, use of dexamethasone, use of nebulization with adrenaline, and ventilatory mode at the time of extubation (spontaneous, SIMV, or A/C). Likewise, the numerical variables were age, weight, tidal volume (Vt), minute volume (Vm), respiratory frequency (RF), partial pressure of oxygen (PaO_2_), FiO_2_, maximum inspiratory pressure (MIP), and PRISM system. Ventilatory parameters prior to extubation were used for the study.

### Statistical analysis

Initially, data entry was carried out in Microsoft Excel 2013. Univariate, bivariate and multivariate analyses were performed in the statistical program STATA 13.0. We worked with a 95%CI and a significance of 5% in the inferential analysis. In the univariate analysis, for the categorical variables, the distributions of absolute and relative frequencies (percentages) were used. Meanwhile, for the numerical variables, measures of central tendency and dispersion were used, such as the mean and standard deviation, and when the data were normally distributed or when it was not normally distributed, the median and interquartile range were used. Likewise, in the bivariate analysis, for the categorical variables, the measure of statistical association was calculated using the chi-squared test (after verification of the assumptions), and for the numerical variables, Student's t-test was used for those with a normal distribution and the Mann-Whitney U test was used for those with a non-normal distribution after confirmation with the Shapiro-Wilk test. For the multivariate analysis, the logistic regression model was used for adjusting the main variables and obtaining the OR; the adjustment was verified using the Hosmer-Lemeshow test.

For the multivariate analysis, the following variables were included: time in ICU, time on MV of greater than 7 days, reason for admission to the ICU, ventilatory mode, use of adrenaline, use of sedatives for greater than 5 days, use of inotropes, and use of dexamethasone. These variables were selected because they were significant in table 1 and/or because of their clinical relevance, since the stability of the patient at the time of extubation can be observed, as well as because of their direct relationship with the outcome (FE) as described by the existing bibliography.^(8,12,13,17-23)^

**Table 1:** Characteristics of cases (failed extubation) and controls (successful extubation)

Variable	Cases (FE) n = 30	Controls (SE) n = 120	p value
Sex†			
Male	18 (60.00)	76 (63.33)	0.73
Age (years)‡	1 (0.3 - 4)	2 (0.2 - 9)	0.29
Weight (kg)	7.15 (4.38 - 15.5)	12 (4.97 - 27.75)	0.09
MV days > 7 days†	24 (80.00)	28 (23.33)	<0.001*
Days in ICU‡	30.5 (13 - 68)	4 (1 - 9.5)	<0.001*
Reason for admission†			0.001*
Respiratory	17 (56.67)	31 (25.83)	
Non-respiratory	13 (43.33)	89 (74.17)	
PRISM‡	9.5 (5 - 16)	9 (5 - 13)	0.34
Respiratory frequency‡	28 (19 - 36)	21 (16 - 30)	0.06
FiO_2_§	33.43 ± 9.76	32.7 ± 8.01	0.66
Tidal volume (mL/kg)‡	15.5 (12 - 85)	34.5 (12-138)	0.48
Volume minute (mL/kg)‡	480 (336-1190)	882 (275-2680)	0.35
PaO_2_§	95.64 ± 43.73	119.99 ± 57.97	0.33
PIP§	17.54 ± 4.72	17,30 ± 3,39	0.75
Ventilation mode prior to extubation†			0.03*
Spontaneous	13 (43.33)	29 (24.17)	
SIMV	9 (30.00)	52 (43.33)	
A/C	7 (23.33)	31 (25.83)	
Inotropic use†	12 (40.00)	65 (54.17)	0.16
Use of sedation ev† > 5 days	21 (70.00)	61 (50.83)	0.05
Use of dexamethasone†	25 (83.33)	82 (68.33)	0.10
Use of nebulized adrenaline†	11 (36.67)	79 (65.83)	0.004*

FE - failed extubation; SE - successful extubation; MV - mechanical ventilation; ICU - intensive care unit; PRISM - pediatric risk of mortality; FiO_2_ - fraction of inspired oxygen; PaO_2_ - partial pressure of oxygen; PIP - peak inspiratory pressure; SIMV - synchronized intermittent mandatory ventilation; A/C - controlled assistance.

* p < 0.05.

† chi-squared test.

‡ Mann-Whitney U test.

§ Student’s t-test. Results are expressed as number (%), median (interquartile range) or mean ± standard deviation.

## RESULTS

**Figure 1:**
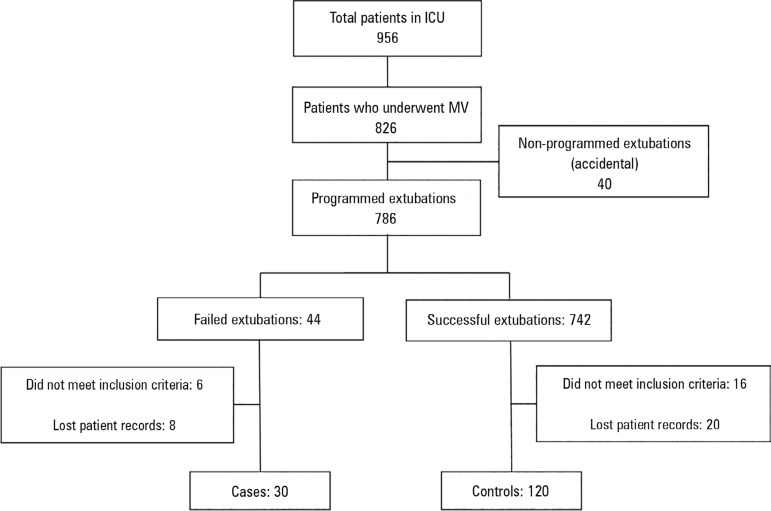
Flow chart of patients. ICU - intensive care unit; MV - mechanical ventilation.

In a period of 5 years, a total of 956 patients were admitted to the ICU, of which 826 were subjected to MV (86%). Of these, 44 patients experienced FE; 6 of them did not meet the inclusion criteria, and 8 records were not located, leaving 30 cases. Of the 742 patients with SE, 16 did not meet the inclusion criteria, and 20 clinical histories were not found; 120 controls were finally used for the study (Figure 1). The proportion of FE was 5.32%. During MV, the study patients were intubated for a total of 1308 days, with a median of 5 and an interquartile range of 1 to 10.

When comparing FE and SE patients according to sociodemographic characteristics, the majority (60%) of the FE patients were boys, and 80% were subjected to prolonged MV, compared to 23.33% for the controls (p < 0.001). Likewise, the length of stay in the ICU was approximately 7 times higher in those with FE (p < 0.001). Respiratory causes (56.67%) were the main reason for hospital admission (p = 0.01). Additionally, it was found that most FE patients were in the spontaneous breathing mode before being extubated, while most of the SE patients were in the SIMV mode prior to extubation (p = 0.03). In addition, the use of nebulized adrenaline was higher (65.83%) in SE patients (p = 0.004). The rest of the results are summarized in table 1.

In the multivariate analysis, the risk factors found for FE included ICU days (OR = 1.04, 95%CI = 1.00 - 1.09, p = 0.03), greater than 7 days of MV (OR = 3.84, 95%CI = 1, 01 - 14.56, p = 0.04) and the use of sedatives for greater than 5 days (OR = 4.81, 95%CI = 1.28 - 18.02, p = 0.02). There was no collinearity between time in the ICU and time on MV (Table 2).

**Table 2:** Results of the multivariate analysis

Independent variables	OR raw	Adjusted OR	IC95%
Time in ICU (days)	1.07	1.04	1.00 - 1.09
VM time > 7 days	13,14	3.84	1.01 - 14.56
Use of sedatives > 5 days	2.25	4.81	1.28 - 18.02
Use of inotropes > 10 days	0.56	0.34	0.10 - 1.12
Use of nebulized adrenaline	0.30	0.34	0.11 - 1.04
Use of dexamethasone	2.31	2.01	0.52 - 7.71
Reason for admission to the ICU	3.75	1.40	0.43 - 4.45
Ventilation mode	0.78	0.98	0.69 - 1.38

OR - odds ratio; 95%CI - 95% confidence interval; ICU - intensive care unit; MV - mechanical ventilation. Adjustments were made between all of the variables.

In addition, the goodness of fit of the logistic regression model was verified through the Hosmer-Lemeshow test. This revealed a value of 0.4345, which verifies that the model is a good fit.

## DISCUSSION

The proportion of patients on MV in the study population was 86%, while the proportion with FE was 5.32%. This result is high compared to other studies performed because the study was conducted in a reference hospital for the pediatric population, which sees complex pathologies and a high prevalence of comorbidities. The other studies of ICU patients found proportions of MV from 30 - 64%^(3,19)^ and proportions of FE from 4-19%.^(24)^ During MV, the study patients were intubated for a total of 1308 days, with a median of 5 and an interquartile range of 1 to 10.

The study found the following factors to be related to FE: times on ventilation greater than 7 days, longer times in the ICU, the use of sedatives for greater than 5 days, reasons for respiratory admission, spontaneous ventilatory modes and the use of nebulized adrenaline.

It was determined that an MV time of greater than 7 days increased the risk of FE by almost 4 times. This result is consistent with the study carried out by Cruces et al., which found that prolonged MV increased the risk of FE by almost 7 times compared to SE.^(16)^ In addition, in the study conducted by Hiremath et al. in 2009, it was found that with prolonged MV, there is a higher risk of FE.^(15)^

In the present study, it was found that patients with FE had longer ICU stays compared to the control group; this was a risk factor. A similar result was found in the study by Gaies et al.,^(25)^ who found that patients with longer ICU stays had a higher risk of developing FE. Additionally, Gupta et al.^(20)^ found that the length of stay in the ICU and the duration of the hospital stay were associated with FE. Additionally, Cruces et al.,^(16)^ in Chile, found an association between time on MV and the length of stay in the ICU.^(16)^

Another associated risk factor was the use of sedatives for greater than 5 days. In a study that evaluated the prediction of FE in pediatric patients, it was observed that patients who received infusions of sedatives for longer periods had a higher risk of FE.^(21)^ In addition, studies of children on MV showed that 27% of patients are oversedated, making it difficult to assess the sensory capacity of the patients and delaying weaning from the ventilator.^(22)^ The conclusions of these studies warn that monitoring of the use of these drugs must be improved to prevent overuse.

Likewise, it was possible to appreciate that respiratory causes (56.67%) were the main reason for hospital admission (p = 0.001) in the cases of FE. In addition, the majority of patients with FE were in the spontaneous breathing mode (43.33%) before being extubated, while the use of the SIMV mode was observed more often among in patients with SE. Studies have been performed on the SIMV ventilatory mode and its associations with a decreased need for sedation, decreased muscle paralysis, and lower number of patients who struggle with the ventilator.^(23)^ This could result in better extubation results and a lower number of FE events.

The study also found that nebulized epinephrine (p = 0.004) was used with 65.83% of the SE patients. According to the study of Davies et al.,^(26)^ theoretically, using nebulized adrenaline can decrease subglottic laryngeal inflammation after extubation, thereby reducing the risk of FE in the pediatric population. However, no positive results have been found, nor is there a consensus on the use of nebulization with adrenaline as an effective routine treatment in the prevention of FE.^(26)^ The most effective dose has not been found; however, an increase in side effects proportional to the dose has been observed.^(27)^

At the end of the multivariate analysis, no association was found between the use of the SIMV ventilatory mode and nebulization with adrenaline as protective factors in extubation. Additionally, in a study conducted by Khemani et al.,^(28)^ it was concluded that there is not enough evidence for the prophylactic use of dexamethasone prior to extubation to prevent FE in children; certain benefits have only been found in the adult population.^(28)^ However, the use of this intervention is common in pediatric intensive care centers in Peru. For this reason, this variable was considered in the study.

The present study has certain limitations as well as a probable measurement bias because the data were not collected directly from patients but were instead extracted from a database and medical records. Likewise, 18.18% of FE cases were lost due to the loss of data. In addition, the inclusion of cases depends on the probability that they were admitted and diagnosed in that hospital. Ventilatory weaning was based on standardized criteria for weaning, while extubation was based on medical clinical judgment.

Accidental extubations (4.84%) is a population group qualitatively different from our study group. This characteristic was used as a restriction criterion, in such a way that this factor was not included, so it does not affect the conclusions.

## CONCLUSION

The present study found that pediatric patients present a higher risk for failed extubation if they spend more time in the intensive care unit, if they are subjected to a prolonged time on mechanical ventilation of greater than 7 days and if they receive sedatives for greater than 5 days.
